# Supplementary material for: A simulation-based analysis of the impact of rhetorical citations in science
Source: Nat Commun. 2024 Jan 10;15:431. doi: 10.1038/s41467-023-44249-0 (PMC10781737; doi:10.1038/s41467-023-44249-0)
Supplement: Supplementary file 1 — Supplementary Information [file 41467_2023_44249_MOESM1_ESM.pdf]

Supplementary Information of
"A simulation-based analysis of the impact of rhetorical citations in science"

Honglin Bao, Misha Teplitskiy

Table of contents

1. Supplementary Discussion
   1.1. Quality and initial rhetorical value
   1.2. Threshold
   1.3. Perception error
   1.4. Fit
   1.5. Reinforcement strengths
2. Supplementary Methods

1. Supplementary Discussion

The Main paper considered several moderation variables, *e.g.*, literature size. Here, as we provide supplementary discussions by varying various parameters to test for robustness, we also parameter-sweep arguably the most important and policy-relevant moderator – the citing budget. As we change the focal parameters, we show how the results vary as the citing budget goes from 20 to 100 and includes more rhetorical citations.

1.1. Varying quality and initial rhetorical value distributions

In the Main model, we assumed long-tail distributions for quality and initial rhetorical value. The tail's fatness may have a significant effect on three metrics of community health. Here, we examine the robustness of our principal conclusions using different value distributions. In the Main model we used β(1, *w*) for distributions of quality and rhetorical value. Parameter *w* determines the fatness of the distribution's tail, which we set *w* at 6. We vary it to 4 and 8 to see how the results will change with more/fewer high-value papers, respectively. In addition, we consider a normal distribution *N*(0.5, 0.1). All distributions are shown in Figure S1, with 10000 draws from each.

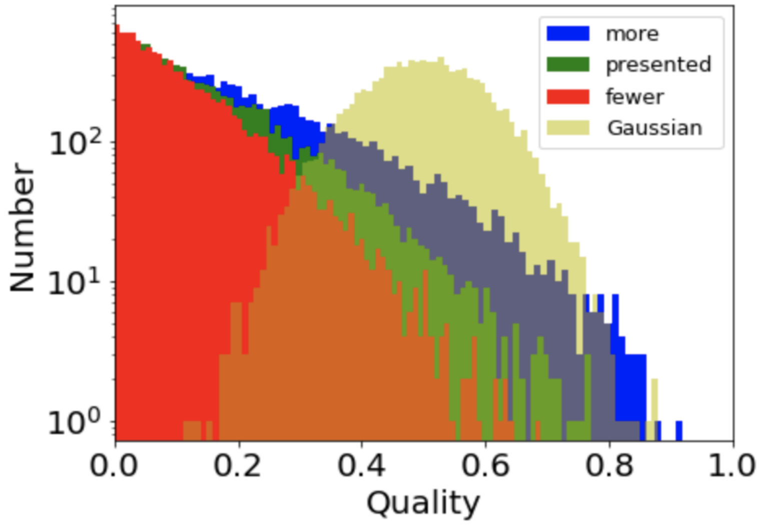

Figure S1. Value distributions. Additional distributions for quality and initial rhetorical value: more high-value papers β(1, 4), fewer high-value papers β(1, 8), and values following a Gaussian distribution *N*(0.5, 0.1) among papers. The presented model in the Main paper uses a Beta distribution β(1, 6) of values with moderate numbers of high-value ones. 10,000 draws from each distribution are displayed.

More high-value papers

Using a distribution with more papers of high quality and rhetorical value ($w = 4$), we find that rhetorical citing still improves the three metrics of health, consistent with the Main results. Figure S2 plots how the metrics for the full and two null models change across different citing budgets. The result for each citing budget is the measurement after a 1000-time-step simulation, averaged by ten random seeds.

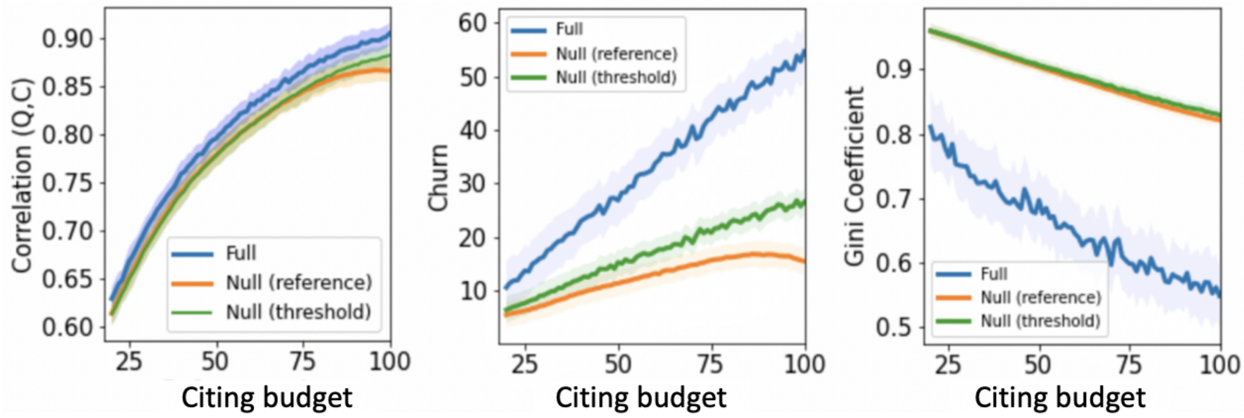

Figure S2. The results with more high-value papers β(1, 4). For each panel, the shaded areas indicate the bootstrapped 95% confidence intervals derived from 20 simulation runs, while the lines depict the average values across these runs.

Fewer high-value papers

Similarly to the above, using a distribution with fewer papers of high quality and rhetorical value ($w = 8$), we find that rhetorical citing still improves the three metrics of health, consistent with the Main results.

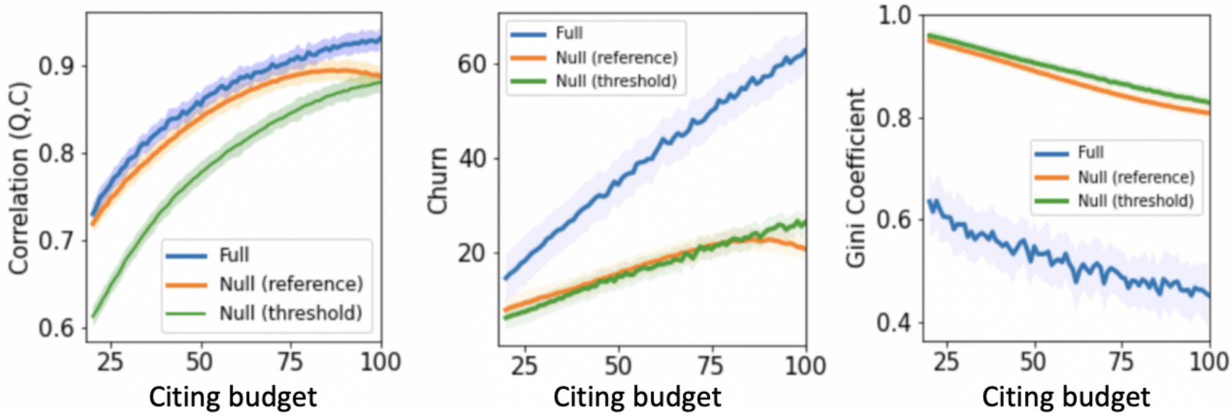

Figure S3. The results with fewer high-value papers β(1, 8). For each panel, the shaded areas indicate the bootstrapped 95% confidence intervals derived from 20 simulation runs, while the lines depict the average values across these runs.

Normal distribution of values

Next, we consider the case where quality and rhetorical value are distributed normally. The full model (with rhetorical citing) improves the metrics relative to the null models, especially for higher citing budgets.

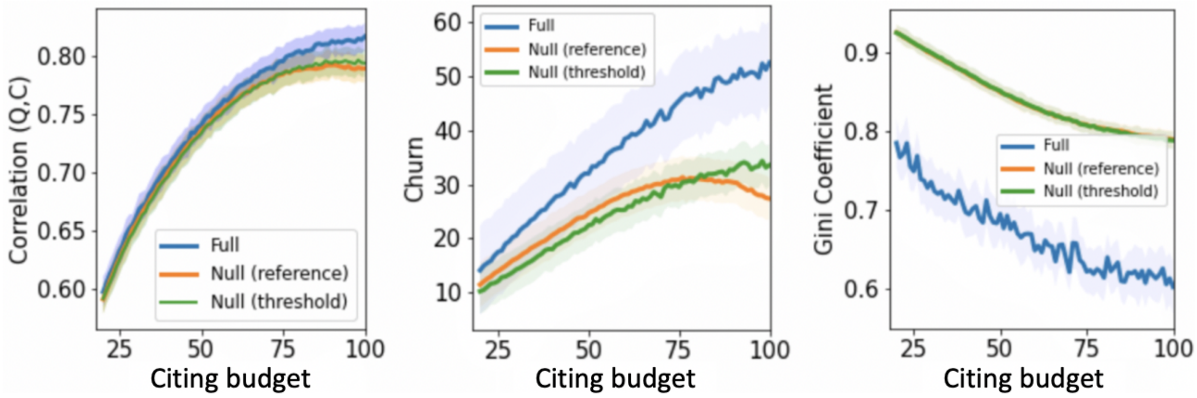

Figure S4. The results under a normal distribution of values $N(0.5, 0.1)$. For each panel, the shaded areas indicate the bootstrapped 95% confidence intervals derived from 20 simulation runs, while the lines depict the average values across these runs.

1.2. Varying distributions of threshold

In the Main results, we sampled adoption thresholds for agents from a uniform distribution on [0,1]. Here, we instead use a truncated normal distribution $N(0.5, 0.2)$ within the range [0,1].

Figure S5 shows that rhetorical citing improves the three metrics, consistent with the Main results.

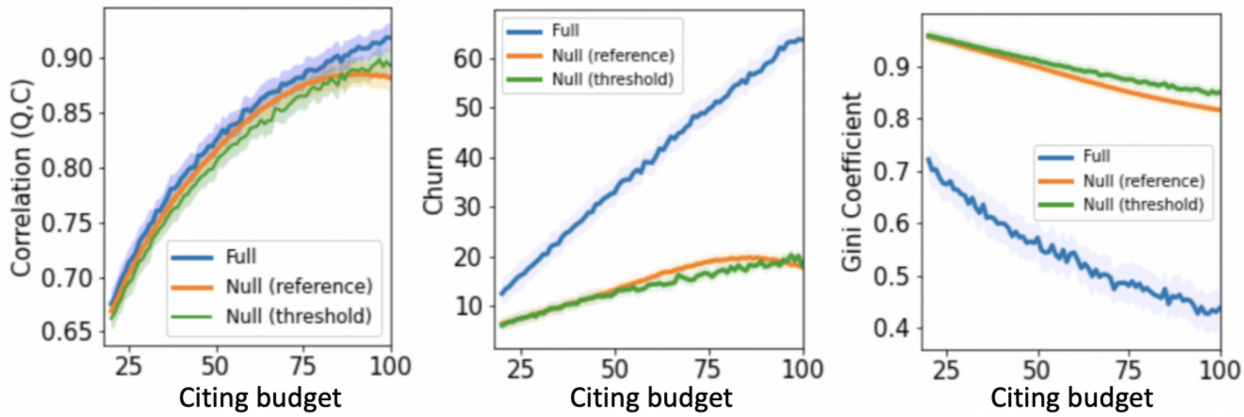

Figure S5. Three models with different thresholds, *N*(0.5, 0.2) truncated within [0,1]. For each panel, the shaded areas indicate the bootstrapped 95% confidence intervals derived from 20 simulation runs, while the lines depict the average values across these runs.

1.3. Varying size of perception error

In the Main result, perception error was distributed *Normal*(0, 0.05). Here, we consider how the results change when the standard deviation is lowered to 0.02 or raised to 0.1.

Higher noise

Figure S6 shows that rhetorical citing improves the three metrics, consistent with the Main results.

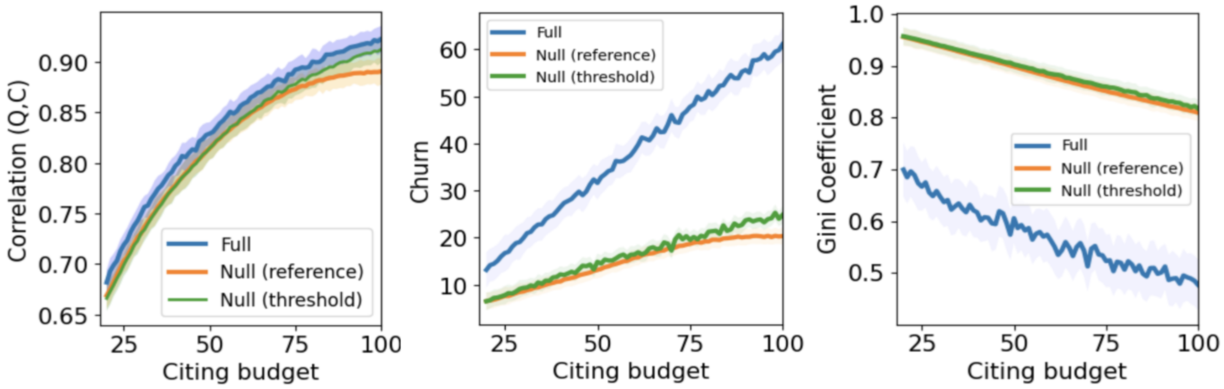

Figure S6. Three metrics of community health with higher perception noise, Normal(0, 0.1). Fit and perception errors can vary a paper's quality from 0.5 to a random number in [0.1,

0.9]. For each panel, the shaded areas indicate the bootstrapped 95% confidence intervals derived from 20 simulation runs, while the lines depict the average values across these runs.

Lower noise

Figure S7 shows that rhetorical citing increases the correlation (left panel), churn (middle panel), and decreases citation inequality (right panel), consistent with the Main results.

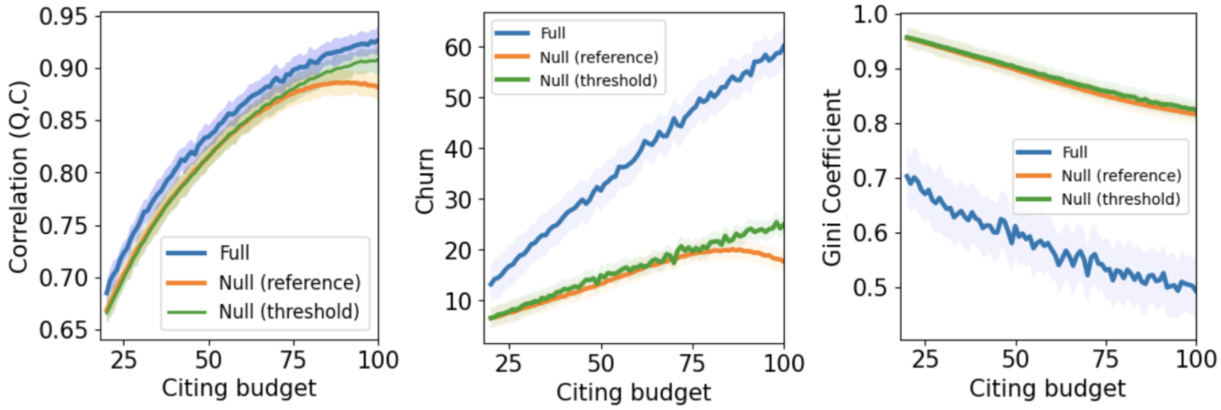

Figure S7. Three metrics of community health with lower perception noise, Normal(0, 0.02). Fit and perception errors can vary a paper's quality from 0.5 to a random number in appx. [0.35, 0.65]. For each panel, the shaded areas indicate the bootstrapped 95% confidence intervals derived from 20 simulation runs, while the lines depict the average values across these runs.

1.4 Varying distribution of *fit*

In the Main result, we sampled *fit* for agents from a uniform distribution on [-0.1,0.1]. Here, we consider how the results change when the range of the distribution is increased to [-0.2,0.2] or decreased to [-0.05,0.05].

Less varied *fit*

Figure S8 shows that rhetorical citing improves the three metrics, consistent with the Main results.

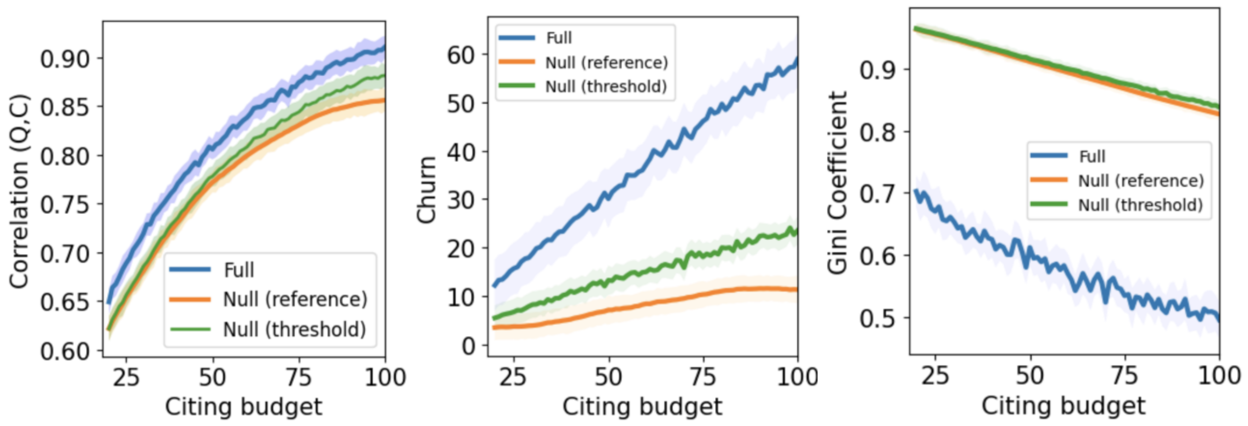

Figure S8. Three metrics of community health with a less varied fit, Uniform(-0.05, 0.05). Fit and perception errors can vary a paper's quality from 0.5 to a random number in [0.3, 0.7]. For each panel, the shaded areas indicate the bootstrapped 95% confidence intervals derived from 20 simulation runs, while the lines depict the average values across these runs.

More varied *fit*

Figure S9 shows that rhetorical citing improves churn (the middle panel) and citation inequality (the right panel). However, the citation-quality correlation changes little between the full and null models. The intuition behind this result is that with a more varied *fit*, existing perceived quality of papers does not much affect whether researchers cite them subsequently as varied fits can significantly raise or lower the quality in readers' eyes. Hence, the lock-in effect observed in the null models in our Main results is reduced. In effect, rhetorical citing has similar but much stronger effects on the community health metrics as adding substantial person-specific *fit*s to the citing process.

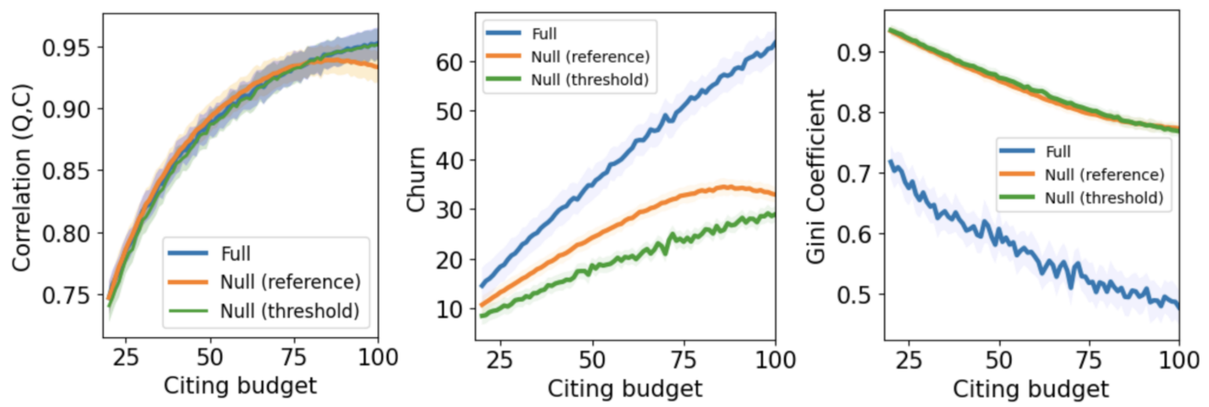

Figure S9. Three metrics of community health with a more varied fit, Uniform(-0.2, 0.2). Fit and perception errors can vary a paper's perceived quality from 0.5 to a random number in

[0.15, 0.85]. For each panel, the shaded areas indicate the bootstrapped 95% confidence intervals derived from 20 simulation runs, while the lines depict the average values across these runs.

1.5 Varying reinforcement strengths

Varing α

The parameter α measures how a paper's citation count affects perceptions of its quality, with $\alpha = 0.001$. Note that because the maximum citation count is 1000, the maximum effect of citations on perceived quality is 0.001*1000 = 1, which equals the maximum underlying quality and maximum underlying rhetorical value. Here, we consider the case $\alpha = 0.002$ (maximum citation premium=2) and $\alpha = 0$ (citations have no impact on perceived quality). Figure S10 shows that the full model outperforms the two null models in the three metrics if readers do not rely on citations to perceive quality. In contrast, Figure S11 shows that under a higher reinforcement strength, the full model outperforms the two null models on churn and inequality, but is similar on correlation. Greater reinforcement increases the concentration of substantive citations among elite-quality papers, and because perceived quality is a component of rhetorical value, increases rhetorical citations on them as well, diminishing the differences between the models.

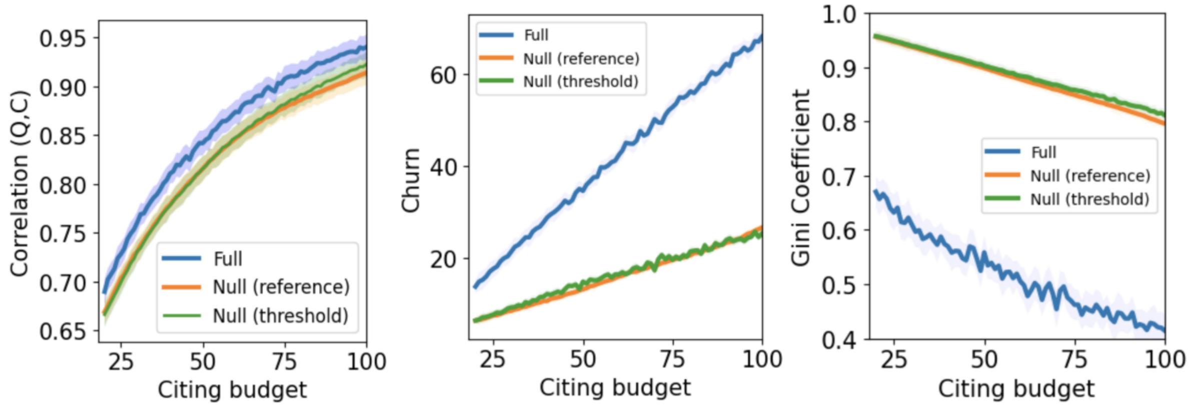

Figure S10. Three metrics under zero reinforcing strength ($\alpha = 0$). For each panel, the shaded areas indicate the bootstrapped 95% confidence intervals derived from 20 simulation runs, while the lines depict the average values across these runs.

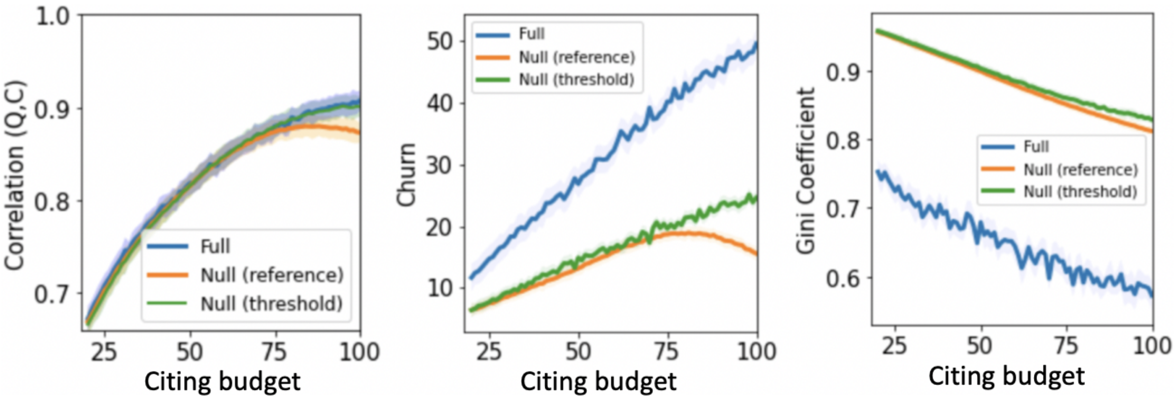

Figure S11. Three metrics under a higher reinforcing strength ($\alpha = 0.002$). For each panel, the shaded areas indicate the bootstrapped 95% confidence intervals derived from 20 simulation runs, while the lines depict the average values across these runs.

Varing β

The parameter β quantifies how a paper's perceived quality affects its rhetorical value, with $\beta = 0.3$ in the Main model. Here we compare the Main model with β = 0, where rhetorical value does not depend on perceived quality at all, and β = 1, where in determining rhetorical value agents place equal weight on how rhetorically useful the paper is to their argument and its perceived quality. Note that because the null models do not have rhetorical citing, we do not include them in this analysis. Figure S11 shows that the full model with β = 0 has the highest citation churn and the lowest citation inequality. The full model with β = 1 has the lowest churn and highest inequality. Interestingly, the model with the intermediate β = 0.3 value that we use in Main has the highest citation-quality correlation. If agents rely on perceived quality very heavily, then the model comes close to the null models without rhetorical citing, lowering the correlation as is observed in the null models. On the other hand, if agents do not rely on perceived quality at all, then the underlying rhetorical value becomes the only driving factor in citing and even low-quality papers receive citations. Thus, very high and very low reinforcement induce lower citations-quality correlations by increasing citing of the upper and lower ends of the quality distribution, respectively.

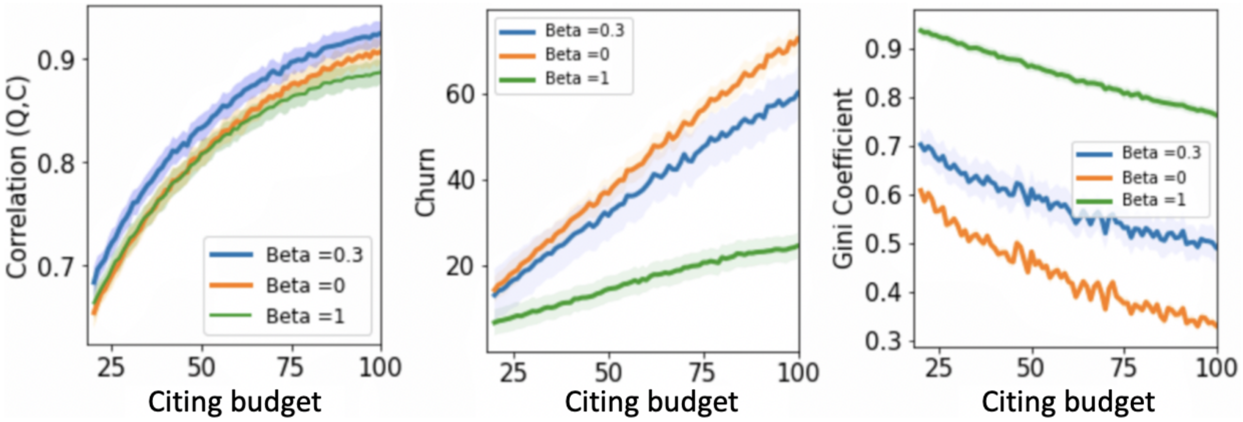

Figure S12. Full models after 1000 iterations with different values of β. For each panel, the shaded areas indicate the bootstrapped 95% confidence intervals derived from 20 simulation runs, while the lines depict the average values across these runs.

2. Supplementary Methods

In contrast to the heterogeneous agent models presented in Main, homogeneous agents perceive quality identically (there is no $fit_{i,j}$), perceive rhetorical value identically, and have the same threshold for adoption 0.5. We initialize models with homogeneous agents the same way as with heterogeneous ones: literature size = 600, reading budget = 120, citing budget = 40, and timesteps = 1000. Figure S12 shows the citation distribution across paper quality. After 1000 iterations, the distribution is nearly bimodal with some papers having 0 citations and some 1000. Given that citation distributions in practice are never bimodal, we do not believe such a simple model is sufficiently realistic. For completeness, we present all three community health metrics for the three versions of this model (Full, Null-reference, Null-threshold) in Figure S13, but do not interpret it further.

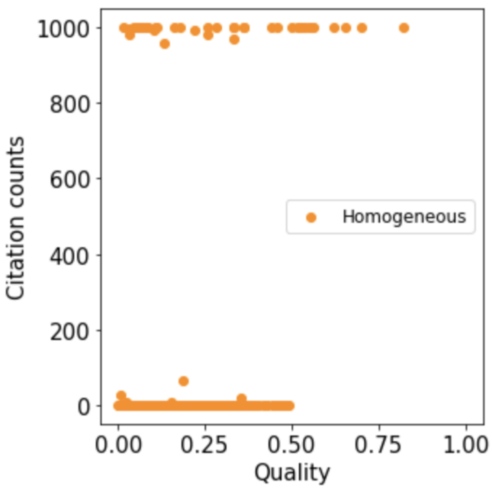

Figure S13. Citation distribution in the homogeneous model after 1000 iterations.

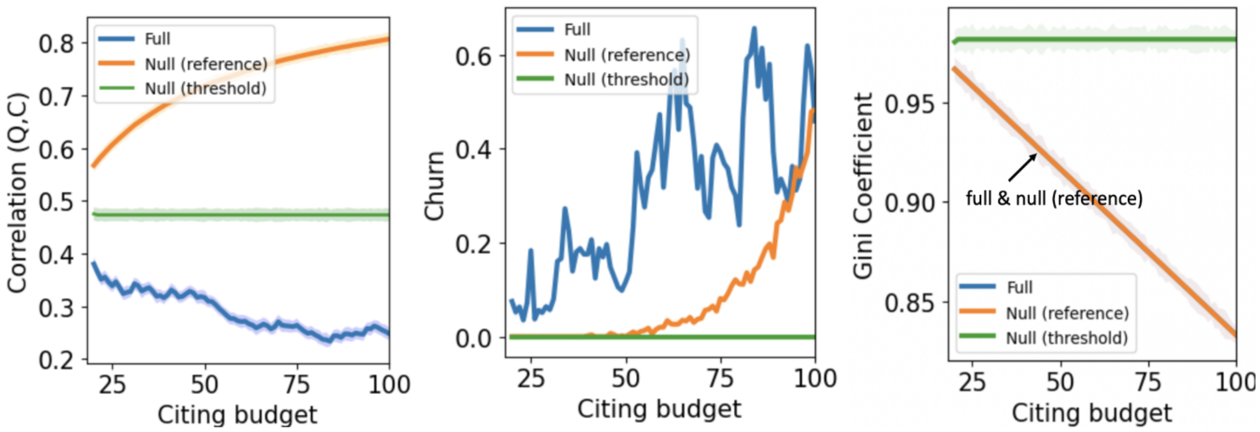

Figure S14. Metrics of community health produced by three models – Full, Null-reference, Null-threshold – all with homogeneous agents, after 1000 iterations.
