# Supplementary material for: A simulation-based analysis of the impact of rhetorical citations in science
Source: Nat Commun. 2024 Jan 10;15:431. doi: 10.1038/s41467-023-44249-0 (PMC10781737; doi:10.1038/s41467-023-44249-0)
Supplement: Supplementary file 3 — Reporting Summary [file 41467_2023_44249_MOESM3_ESM.pdf]

nature portfolio

Corresponding author(s): Misha Teplitskiy, PhD

Last updated by author(s): Oct 27, 2023

Reporting Summary

Nature Portfolio wishes to improve the reproducibility of the work that we publish. This form provides structure for consistency and transparency in reporting. For further information on Nature Portfolio policies, see our Editorial Policies and the Editorial Policy Checklist.

Statistics

For all statistical analyses, confirm that the following items are present in the figure legend, table legend, main text, or Methods section.

n/a	Confirmed	
☐	☒	The exact sample size (*n*) for each experimental group/condition, given as a discrete number and unit of measurement
☐	☒	A statement on whether measurements were taken from distinct samples or whether the same sample was measured repeatedly
☐	☒	The statistical test(s) used AND whether they are one- or two-sided<br>*Only common tests should be described solely by name; describe more complex techniques in the Methods section.*
☐	☒	A description of all covariates tested
☐	☒	A description of any assumptions or corrections, such as tests of normality and adjustment for multiple comparisons
☐	☒	A full description of the statistical parameters including central tendency (e.g. means) or other basic estimates (e.g. regression coefficient) AND variation (e.g. standard deviation) or associated estimates of uncertainty (e.g. confidence intervals)
☐	☒	For null hypothesis testing, the test statistic (e.g. *F*, *t*, *r*) with confidence intervals, effect sizes, degrees of freedom and *P* value noted<br>*Give P values as exact values whenever suitable.*
☒	☐	For Bayesian analysis, information on the choice of priors and Markov chain Monte Carlo settings
☒	☐	For hierarchical and complex designs, identification of the appropriate level for tests and full reporting of outcomes
☐	☒	Estimates of effect sizes (e.g. Cohen's *d*, Pearson's *r*), indicating how they were calculated

Our web collection on statistics for biologists contains articles on many of the points above.

Software and code

Policy information about availability of computer code

Data collection	no data collection since this is a theoretical simulation study.
Data analysis	All data generation and analysis were performed using custom Python 3.10.0 code, without the reliance on additional software.

For manuscripts utilizing custom algorithms or software that are central to the research but not yet described in published literature, software must be made available to editors and reviewers. We strongly encourage code deposition in a community repository (e.g. GitHub). See the Nature Portfolio guidelines for submitting code & software for further information.

Data

Policy information about availability of data

All manuscripts must include a data availability statement. This statement should provide the following information, where applicable:

- Accession codes, unique identifiers, or web links for publicly available datasets
- A description of any restrictions on data availability
- For clinical datasets or third party data, please ensure that the statement adheres to our policy

The simulation code for all models and data generated in this study have been deposited in a persistent GitHub repository https://github.com/hlbao/rhetorical_citing (DOI: 10.5281/zenodo.10038833). We have included the statement (plus software usage) in the paper.

Research involving human participants, their data, or biological material

Policy information about studies with human participants or human data. See also policy information about sex, gender (identity/presentation), and sexual orientation and race, ethnicity and racism.

Reporting on sex and gender	na since this is a theoretical simulation study.
Reporting on race, ethnicity, or other socially relevant groupings	na since this is a theoretical simulation study.
Population characteristics	na since this is a theoretical simulation study.
Recruitment	na since this is a theoretical simulation study.
Ethics oversight	na since this is a theoretical simulation study.

Note that full information on the approval of the study protocol must also be provided in the manuscript.

Field-specific reporting

Please select the one below that is the best fit for your research. If you are not sure, read the appropriate sections before making your selection.

☐ Life sciences ☒ Behavioural & social sciences ☐ Ecological, evolutionary & environmental sciences

For a reference copy of the document with all sections, see nature.com/documents/nr-reporting-summary-flat.pdf

Behavioural & social sciences study design

All studies must disclose on these points even when the disclosure is negative.

Study description	This paper uses a family of agent-based models to show the beneficial effects of rhetorical citations (citing without being inspired): they redistribute focus from elite papers to high-to-mid-quality ones and benefit academic community health by reducing the Matthew Effect. Quantitative data were generated in the simulation.
Research sample	We use simulation modeling, a theoretical approach, so we do not have research samples.
Sampling strategy	This is simulation modeling, so no sample size calculation was performed.
Data collection	simulated data, researchers were blinded to the study hypothesis. No random allocation of experimental vs. control conditions.
Timing	2021 Aug to 2022 May
Data exclusions	no data were excluded
Non-participation	no participants dropped out
Randomization	No random allocation of experimental vs. control groups was performed since this is a simulation study.

Reporting for specific materials, systems and methods

We require information from authors about some types of materials, experimental systems and methods used in many studies. Here, indicate whether each material, system or method listed is relevant to your study. If you are not sure if a list item applies to your research, read the appropriate section before selecting a response.

Materials & experimental systems

n/a	Involved in the study	
☒	☐	Antibodies
☒	☐	Eukaryotic cell lines
☒	☐	Palaeontology and archaeology
☒	☐	Animals and other organisms
☒	☐	Clinical data
☒	☐	Dual use research of concern
☒	☐	Plants

Methods

n/a	Involved in the study	
☒	☐	ChIP-seq
☒	☐	Flow cytometry
☒	☐	MRI-based neuroimaging

Plants

Seed stocks	no plants involved
Novel plant genotypes	no plants involved
Authentication	no plants involved
